# Literary Notes

**Published:** 1899-06

**Authors:** 


### LITERARY NOTES.

THE Michigan State Board of Health has recently issued an official publication, setting forth by photographs, of views throughout the state, the facts showing the desirability of the state as a summer resort, and its value as a health resort. This publication should be of interest to many persons in the sister states on the south; and the publication sets out attractions that appeal to persons who are in persuit of health or pleasure in the way of sailing, rowing, angling and the enjoyment of picturesque and pleasant surroundings.

The Directory can be obtained by sending six cents in stamps to pay postage, with your request, and name and address to State Board of Health, Lansing, Michigan.

DR. A. A. HUBBELL, of Buffalo, published in volume second of the *Iris*, 1899, an interesting historical sketch of the Medical Department of Niagara University. The JOURNAL is indebted to the author for a reprint.

THE *Jeffersonian* is the name of a new periodical, published by the students of Jefferson Medical College, Philadelphia. The editors desire Jefferson alumni to communicate their addresses to them at the college.

---

DR. JOHN H. HOLLISTER, of Chicago, after many years of service in the editorial chair, has retired from the *Practitioner*, and his magazine has been consolidated with the *Medical Standard.—American Medical Journalist.*

